# Subcutaneous rituximab in patients with diffuse large B cell lymphoma and follicular lymphoma: Final results of the non‐interventional study MabSCale

**DOI:** 10.1002/cam4.5160

**Published:** 2022-08-26

**Authors:** Jan Dürig, Jens Uhlig, Anke Gerhardt, Markus Ritter, Gunnar Hapke, Jörg Heßling, Peter Staib, Frieder Wolff, Katja Krumm, Ludwig Fischer von Weikersthal

**Affiliations:** ^1^ Department of Hematology University Medicine Essen Essen Germany; ^2^ Oncological Practice Naunhof Germany; ^3^ Medical Care Centre for Blood and Cancer Diseases Potsdam Germany; ^4^ Department of Hematology and Oncology Hospital Sindelfingen‐Böblingen Germany; ^5^ Marienkrankenhaus Hamburg Hamburg Germany; ^6^ Onkologie am Segelfliegerdamm Berlin Germany; ^7^ St.‐Antonius Hospital Eschweiler Germany; ^8^ Roche Pharma AG, Biometrics & Epidemiology Grenzach‐Wyhlen Germany; ^9^ Roche Pharma AG, Department of Hematology Grenzach‐Wyhlen Germany; ^10^ St. Marien Health Centre Amberg Germany

**Keywords:** complete response, diffuse large B cell lymphoma (DLBCL), follicular lymphoma, non‐interventional study, progression‐free survival, subcutaneous rituximab

## Abstract

**Background:**

Rituximab has become a standard treatment for non‐Hodgkin lymphoma. Clinical studies have demonstrated the efficacy of rituximab in combination with standard chemotherapies in the treatment of follicular lymphoma (FL) and diffuse large B cell lymphoma (DLBCL) patients. This non‐interventional study aimed to evaluate the effectiveness and safety of subcutaneous (SC) rituximab in routine clinical practice.

**Methods:**

Adult patients with previously untreated CD20 positive DLBCL or FL who received rituximab SC and chemotherapy as first‐line treatment were observed between 07/2014 and 07/2019 at 99 institutions in Germany. Primary endpoint was the (unconfirmed) complete remission (CR/CRu) rate. Primary outcome was analyzed inferentially; other variables were evaluated descriptively.

**Results:**

Overall 583 patients (247 FL; 336 DLBCL) were evaluated. CR/CRu rates were 51.4% (95% CI: 45.2; 57.6) in the FL set and 48.5% (95% CI: 43.2; 53.8) in the DLBCL set. Regarding progression‐free survival in the FL group, the probability of being event‐free was 94.2% in the first year and 86.2% in the second year. An overall response was achieved in 85.8% (FL) and 85.4% patients (DLBCL). Patient satisfaction at the end of study with the time saving simplification of the SC vs. intravenous route was 98% for FL and 97% for DLBCL. 45.3% of FL and 47.0% of DLBCL patients experienced an adverse event of grade ≥3. Serious adverse events of grade ≥3 occurred in 27.9% FL and 32.4% DLBCL patients, with the highest incidences for leucopenia, anemia, nausea, and fatigue. No new safety signals were detected.

**Conclusions:**

The results confirmed the effectiveness and safety of rituximab SC in both the FL and the DLBCL group. Satisfaction of patients and nurses with SC administration was high.

## Graphical abstract

The non‐interventional study MabSCale assessed the effectiveness and safety of subcutaneous rituximab for the treatment of follicular lymphoma (FL) and diffuse large B cell lymphoma (DLBCL). The results confirm the effectiveness and safety of subcutaneous rituximab in both the FL and the DLBCL group. Furthermore, patient and nurse satisfaction with subcutaneous administration was high.

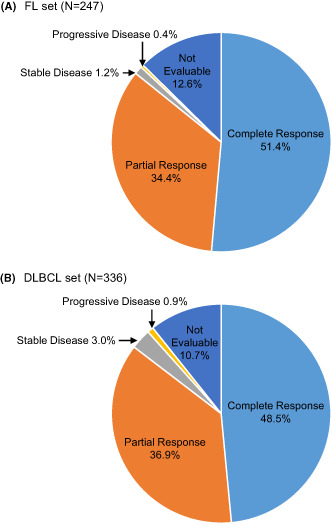

## 1 INTRODUCTION

Diffuse large B cell lymphoma (DLBCL) is the most frequent subtype of non‐Hodgkin lymphoma (NHL) and accounts for up to 40% of cases worldwide.^1^ With approximately 22% of new cases of B cell NHL diagnosed, more than 18,000 people are affected by this condition each year in the US.^2^ In Germany, the incidence is around seven cases per 100,000 annually, with slightly more men affected than women.^3^ While this malignancy is heterogeneous and aggressive, scientific progress over the last quarter century have turned it largely curable with a dual therapeutic approach combining chemotherapy and immunotherapy. The immunotherapy of choice is rituximab administered intravenously (IV) or subcutaneously (SC).^4^, ^5^, ^6^, ^7^

Of the approximately 81,560 cases of NHL expected to be diagnosed in 2021 in the US, roughly 20% are likely to be follicular lymphomas (FL).^8^ In Germany and neighboring states, follicular lymphoma accounts for 20%–35% of all newly diagnosed cases of NHL, with the median age of morbidity between 60 and 65 years.^9^ Typically, no sex preponderance is observed in follicular lymphomas; however, the incidence increases with age and varies across racial groups. Current data from the anti‐CD20 era indicated a median overall survival of up to 20 years.^10^, ^11^ As with DLBCL, multi‐agent chemotherapy in combination with rituximab is the most common first‐line treatment strategy for follicular lymphoma.

Rituximab is a human/murine chimeric monoclonal antibody with specific affinity for the B‐lymphocyte transmembrane protein CD20. Upon binding of CD20+ cells, rituximab induces complement and antibody‐dependent cellular cytotoxicity and has direct anti‐proliferative activity and apoptosis‐inducing effects in tumor cells. With the marketing authorization in 1998, the application of therapeutic antibodies in the treatment of NHL was established. Randomized clinical trials have shown the efficacy and safety of rituximab as monotherapy and in combination with chemotherapy in patients with indolent and aggressive forms of NHL.^12^, ^13^, ^14^ Standard chemotherapy regimens used with rituximab include cyclophosphamide, doxorubicin, vincristine, and prednisone (CHOP) for DLBCL; and CHOP, cyclophosphamide, vincristine and prednisone (CVP) or bendamustine for FL.^13^, ^15^, ^16^, ^17^

While rituximab is conventionally administered intravenously (typically over 2.5 h), a subcutaneous formulation has been developed, reducing healthcare resource burden without compromising clinical activity. Studies in patients with follicular lymphomas showed comparable serum levels when a SC dose of 1400 mg rituximab was administered, compared with the standard IV dose of 375 mg/m^2^ ^18^, ^19^ leading to widespread approval of rituximab SC in Europe and several other countries. The clinical development and evaluation of rituximab SC is well documented in four clinical trials, namely SPARKTHERA, SABRINA, MabEASE, and PrefMab.^7^, ^20^, ^21^, ^22^

The present non‐interventional study (NIS) MabSCale aimed to observe responsiveness, effectiveness, and tolerability of rituximab SC in patients in both main indications, DLBCL and follicular lymphoma and to supplement the rituximab SC marketing authorization data by observing clinical practice.

## 2 PATIENTS AND METHODS

### 2.1 Patients and study design

This non‐comparative, multi‐center NIS prospectively collected data on effectiveness, safety, and utilization of rituximab SC (MabThera SC®) in patients with malignant lymphoma (DLBCL or FL) in routine clinical practice. The target population were adult patients with untreated CD20 positive DLBCL or FL who received first‐line treatment with rituximab SC combined with chemotherapy according to the German label and the summary of product characteristics (SmPC, 20). Patients were excluded if they had contraindications, interactions or incompatibilities to rituximab SC (according to SmPC), were pregnant or breastfeeding women. Retrospective documentation was permitted up to 6 weeks after initiation of therapy. All available patients who gave signed informed consent and who received at least one dose of rituximab SC in routine clinical practice between 08 July 2014 (start of data collection) and 22 July 2019 were included.

The intended observation period was 6 months for DLBCL patients and 30 months for FL patients according to standard treatment of basic disease.

All German sites treating patients with DLBCL or FL could participate. The study protocol was approved by the ethics committee of Landesärztekammer Baden‐Württemberg, Germany, on 03 December 2013. The study was registered under clinicaltrials.gov identifier NCT02240316.

### 2.2 Endpoints and data sources

Primary study endpoint was the (unconfirmed) complete remission (CR/CRu) rate. CR/CRu rate at the end of induction (EOI) and the end of study (EOS) was performed as sensitivity analysis to match previous analyses. The examination of tumor response was carried out according to clinical practice. Secondary endpoints were 2‐year PFS for FL patients with maintenance therapy, the safety profile of rituximab SC in induction (all patients) and maintenance therapy (only FL patients) and best overall response. Due to the different temporal course of DLBCL and FL, endpoints varied between these groups.

Exploratory endpoints included the treatment schedule and treatment duration, chemotherapy regimen and the patients' satisfaction with rituximab SC therapy assessed by the nurses. Post hoc, the study descriptively assessed differences in treatment modalities and response in hospital settings compared to private practice (including medical care centers).

For the assessment of patient satisfaction, the nurses answered the following four questions using predefined response options in the eCRF: (i) In your opinion, how did the patient feel about the subcutaneous administration compared to the IV administration? (1 = as a time‐saving simplification, 2 = no difference to IV administration, 3 = unsafe, 4 = uncomfortable); (ii) What is your impression of the local tolerability of subcutaneous administration? (1 = patient tolerated subcutaneous administration without any problems—no abnormalities, 2 = patient tolerated subcutaneous administration well—mild transient asymptomatic skin reaction, 3 = patient did not tolerate subcutaneous administration well—significant, symptomatic skin reaction, 4 = patient experienced pain during application); (iii) How do you assess the benefit for the patient? (1 = very high, 2 = high, 3 = no benefit, 4 = negative); (iv) How satisfied is the patient with the injection under the skin? (1 = very highly satisfied, 2 = highly satisfied, 3 = neutral, 4 = unsatisfied, 5 = very dissatisfied).

In addition, the nurses assessed subcutaneous administration compared with IV administration with respect to the following three aspects: (i) Complicating the nurse's tasks (1 = yes, 0 = no, 2 = not assessable); (ii) Avoiding infusion rush‐hour (1 = yes, 0 = no, 2 = not assessable); (iii) Easing procedures at the site (1 = yes, 0 = no, 2 = not assessable).

Study‐relevant data were derived from patient files at the participating site. All patient data were recorded on electronic case report forms by the physician or by a person authorized by the physician.

### 2.3 Statistical analysis

The primary outcome CR/CRu rate was analyzed inferentially displaying estimation rates with 95% CIs (Wilson score). Kaplan–Meier analyses were provided for the secondary endpoint PFS. The presentation of all other variables was done descriptively using summary statistics. The analysis of safety variables was based on the incidence and severity of all adverse events (AEs), serious adverse events (SAEs) and AEs with National Cancer Institute (NCI) Common Terminology Criteria for Adverse Events (CTCAE) grade (version 4.0). AEs were coded according to the Medical Dictionary for Regulatory Activities (MedDRA) version 22.0.

Empty data fields in the eCRF were generally treated as missing values. No assessment or replacement of outliers was performed. All analyses were performed for the all‐treated set (*N* = 583) stratified by indication group.

## 3 RESULTS

### 3.1 Patient disposition and baseline characteristics

Overall, 689 potential patients were screened at 99 study centers across Germany (Figure S1). Of these, 668 patients were enrolled and 583 patients with at least one dose of study medication were included in the all‐treated set (Figure S2).

Of all treated patients, 126/247 (51.0%) FL patients and 242/336 (72.0%) DLBCL patients remained on rituximab SC until the end of study. Conversely, 116 (47.0%) FL patients and 92 (27.4%) DLBCL patients, excluding patients with missing final examination, discontinued treatment with rituximab SC prematurely (drop‐outs). The most frequent specific reason cited for study discontinuation in the FL group was “investigator's decision” in 37/116 (31.9%) patients and “disease progression” in 16/92 (17.4%) DLBCL patients. In the FL group, 19/116 (16.4%) patients discontinued due to “disease progression.” Remarkably, only 51/99 and 61/99 study centers treated FL or DLBCL patients, respectively, for the entire study duration.

The demographic and clinical characteristics of the study groups are detailed in Table 1. The median age in the FL group was 65 years and in the DLBCL group 68 years. Gender distribution was balanced in the FL group (49% men, 51% women), while the DLBCL group included a slightly higher proportion of men (57.7%).

**TABLE 1:** Baseline characteristics in the FL and DLBCL set

	FL set (*N* = 247)	DLBCL set (*N* = 336)
Median age (range) [years]	65.0 (26.0; 90.0)	68.0 (24.0; 90.0)
Gender [*n* (%)]		
Men	121 (49.0%)	194 (57.7%)
Women	126 (51.0%)	142 (42.3%)
ECOG performance status [*n* (%)]	(*N =* 242)^a^	(*N =* 324)^a^
≥1	87 (36.0%)	161 (49.7%)
Serum LDH level [*n* (%)]	(*N =* 245)^a^	(*N =* 332)^*a*^
135–225 U/L	148 (59.9%)	151 (44.9%)
Outside norm	97 (39.3%)	181 (53.9%)
Comorbidities^b^ [*n* (%)]	178 (72.1%)	259 (77.1%)
Vascular disorders	107 (43.3%)	156 (46.4%)
Metabolism and nutrition disorders	48 (19.4%)	80 (23.8%)
Endocrine disorders	42 (17.0%)	37 (11.0%)
Neoplasms benign, malignant and unspecified	37 (15.0%)	54 (16.1%)
Cardiac disorders	34 (13.8%)	58 (17.3%)
Surgical and medical procedures	26 (10.5%)	47 (14.0%)
Tumor status [*n* (%)]		
CD20 positive [*n* (%)]	245 (99.2%)	336 (100%)
Bulky‐disease [*n* (%)]		
Yes [*n* (%)]	—	61 (18.2%)
Radiotherapy planned [*n* (%)]		(*N =* 61)^a^
Yes [*n* (%)]	—	31 (9.2%)
GELF criteria [*n* (%)]	(*N =* 131)^a^	
Tumor >7 cm [*n* (%)]	43 (17.4%)	—

Abbreviations: DLBCL, diffuse large B cell lymphoma; ECOG, Eastern Cooperative Oncology Group; FL, follicular lymphoma; GELF, Groupe d'Etude des Lymphomas Folliculaires; LDH, lactate dehydrogenase.

^a^ Patients with data available.

^b^ Patients with at least one comorbidity and an incidence rate >10% per system organ class in both groups.

In the FL group, most patients (47.8%) were diagnosed with grade 2 FL and advanced Ann Arbor tumor status III (33.2%) or IV (40.5%) (Table 2). The FL‐specific international prognostic index (FLIPI) indicated low/intermediate/high risk for 24.7%/32.0%/43.3% of patients. In the DLBCL group, 99.1% of the patients were diagnosed with CD20 positive DLBCL; 3 (0.9%) patients were classified as FL 3b (Table 2). About half of the DLBCL patients had Ann Arbor tumor status I (28.6%) or II (24.7%). The prognostic IPI classification of DLBCL patients with low/low‐intermediate/high‐intermediate/high was 37.8%/36.9%/18.2%/7.1%.

**TABLE 2:** Tumor characteristics at baseline in the FL and DLBCL set and subgroups by type of site (hospital vs. practice)

FL set	Total (*N* = 247)	Hospitals (*N* = 104)	Practices (*N* = 143)
Ann Arbor^a^ tumor status			
I	28 (11.3%)	11 (10.6%)	17 (11.9%)
II	37 (15.0%)	10 (9.6%)	27 (18.9%)
III	82 (33.2%)	38 (36.5%)	44 (30.8%)
IV	100 (40.5%)	45 (43.3%)	55 (38.5%)
FLIPI^b^			
Low risk	61 (24.7%)	24 (23.1%)	37 (25.9%)
Intermediate risk	79 (32.0%)	33 (31.7%)	46 (32.2%)
High risk	107 (43.3%)	47 (45.2%)	60 (42.0)
Grade of FL			
Grade 1	53 (21.5%)		
Grade 2	118 (47.8%)		
Grade 3a	59 (23.9%)		
Not applicable	17 (6.9%)		

DLBCL set	Total (*N* = 336)	Hospitals (*N* = 165)	Practices (*N* = 171)
Ann Arbor^a^ tumor status	(*N* = 335)^c^		(*N* = 170)^c^
I	96 (28.6%)	39 (23.6%)	57 (33.5%)
II	83 (24.7%)	39 (23.6%)	44 (25.9%)
III	62 (18.5%)	30 (18.2%)	32 (18.8%)
IV	94 (28.0%)	57 (34.5%)	37 (21.8%)
IPI^d^			
Low risk	127 (37.8%)	60 (36.4%)	67 (39.2%)
Low‐intermediate risk	124 (36.9%)	60 (36.4%)	64 (37.4%)
High‐intermediate risk	61 (18.2%)	31 (18.8%)	30 (17.5%)
High risk	24 (7.1%)	14 (8.5%)	10 (5.8%)
Type of aggressive lymphoma			
DLBCL	333 (99.1%)		
FL 3b	3 (0.9%)		

Abbreviations: DLBCL, diffuse large B cell lymphoma; FL, follicular lymphoma; FLIPI, Follicular Lymphoma International Prognostic Index; IPI, International Prognostic Index.

^a^ According to.^23^

^b^ According to.^24^

^c^ Number of patients with data available.

^d^ According to.^25^

### 3.2 Primary effectiveness endpoint—CR/CRu rates

The analysis of the primary endpoint (documented CR status at least once during the study) resulted in CR/CRu rates of 51.4% (95% CI: 45.2; 57.6) for the FL set and 48.5% (95% CI: 43.2; 53.8) for the DLBCL set.

In order to facilitate the comparison with previous studies, a sensitivity analysis was performed, including patients with documented CR status at end of induction (EOI) or end of study (EOS). EOI phase was defined as completed staging visit for FL patients, and end of study was defined as a completed end of study visit (FL and DBCL patients).

For FL patients, CR/CRu rates of 34.4% (95% CI: 28.8; 40.5) at EOI and 37.2% (95% CI: 31.5; 43.4) at EOS were achieved (Table 3). In the DLBCL set, the CR/CRu rate was 44.3% (95% CI: 39.1; 49.7) at EOS.

**TABLE 3:** CR/CRu rate: sensitivity analysis in the FL and DLBCL set

Analysis period	CR/CRu achieved	FL (*N* = 247)	DLBCL (*N* = 336)
Induction phase^a^	CR/CRu, *n* (%)	85 (34.4%)	NA
	Rate [95% CI]	34.4 [28.8; 40.5]	NA
End of study^b^	CR/CRu, *n* (%)	92 (37.2%)	149 (44.3%)
	Rate [95% CI]	37.2 [31.5–43.4]	44.3 [39.1–49.7]

Abbreviations: CI, Wilson confidence interval; CR, complete remission; CRu, complete remission unconfirmed; DLBCL, diffuse large B cell lymphoma; FL, follicular lymphoma; NA, not applicable.

^a^ End of induction phase defined as complete staging visit.

^b^ Defined as complete end of study visit.

In addition, subgroup analyses were performed for the primary outcome regarding the following parameters: age (FL [<65; 65 to <75; ≥75], DLBCL [<60; 60 to <80; ≥80]), gender, Eastern Cooperative Oncology Group (ECOG) performance status (0, 1, 2, 3, 4) and Ann Arbor tumor status (I, II, III, IV) (Table S1). Regarding these analyses, the best response rate was reached in the age groups <65 years for FL and < 60 years for DLBCL with CR/CRu rates of 62.8% and 56.7%, respectively. There were no obvious gender differences in either group.

### 3.3 Secondary effectiveness endpoints—progression‐free survival and best overall response

**FIGURE 1:**
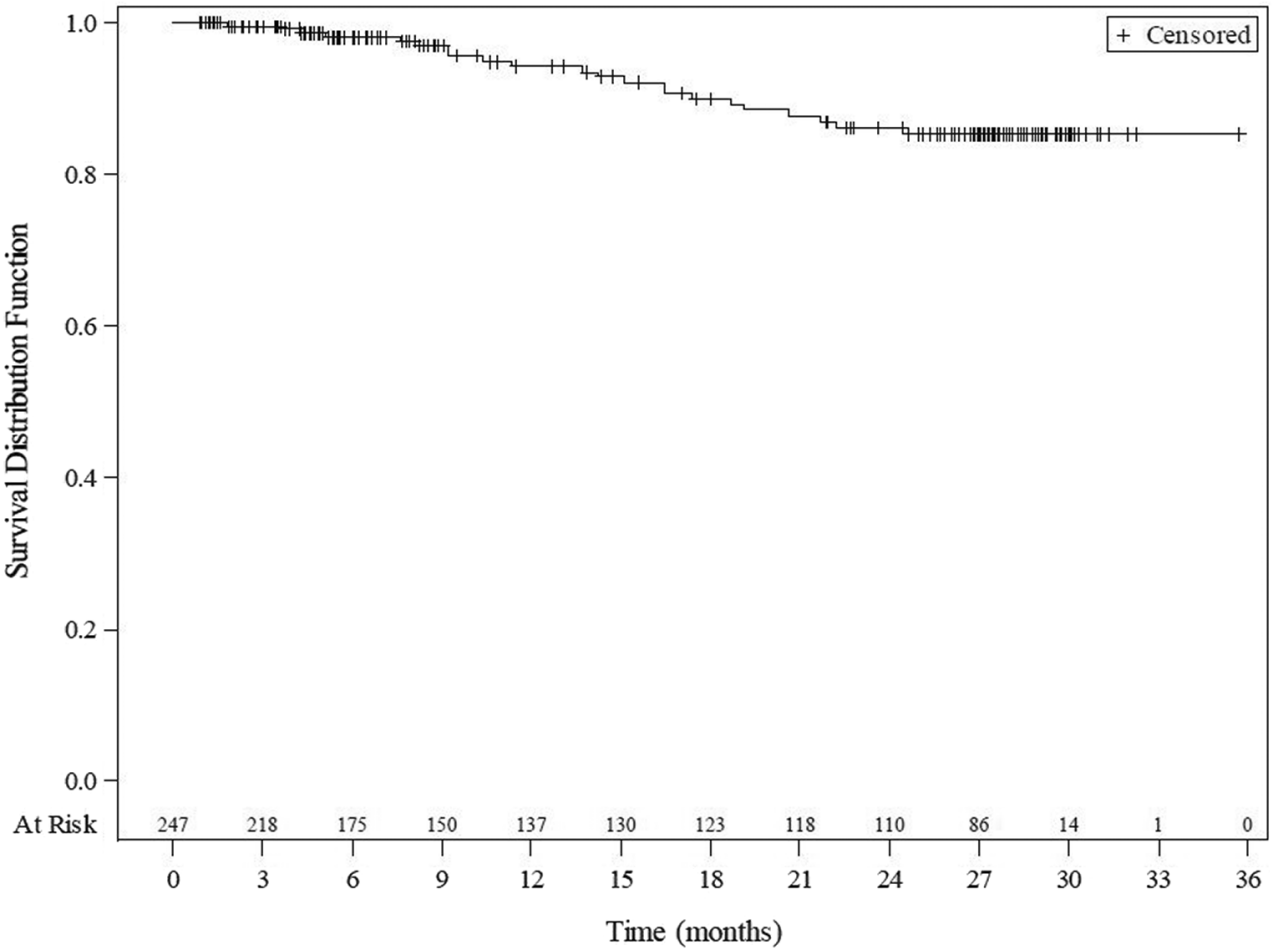
Progression‐free survival in the FL set. Kaplan–Meier plot, patients without event were censored at their last observation (last visit or discontinuation). FL, follicular lymphoma.

A secondary effectiveness variable in the FL group was the 2‐year PFS rate defined as time from first visit until progression or death. The probability of being event‐free in the first year was 94.2% and 86.2% in the second year. Only 22/247 (8.9%) patients had an event. The median PFS time in the FL group has not been reached (Figure 1, Figure S3).

**FIGURE 2:**
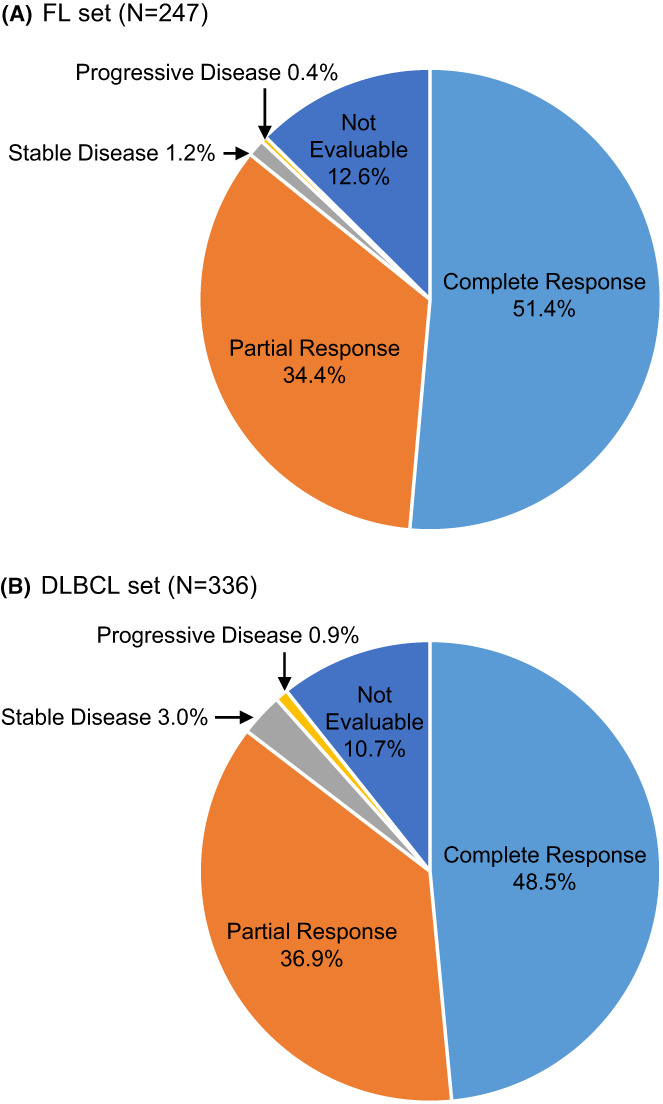
Best overall response. Pie charts show the proportion of patients with complete response, partial response, stable disease, progressive disease, and of non‐evaluable patients in the FL (A) and DLBCL set (B). DLBCL, diffuse large B cell lymphoma; FL, follicular lymphoma.

In the all‐treated set, an overall response (complete [CR] or partial response [PR]) was achieved in 85.8% (FL) and 85.4% (DLBCL) of the patients (Figure 2). In the FL group, the best overall responses (BORs) were achieved at the staging visit (CR 44.9%, PR 53.5%) and end of study (closing visit; CR 79.1%, PR 17.6%). For DLBCL, the response rates at the end of study (closing visit) were 62.4% (CR) and 32.1% (PR), respectively. Progressive disease (PD) rates below 1% further support the effectiveness of the treatment.

### 3.4 Treatment modalities

The median overall treatment duration in the FL group was 17.1 months (range: 0–35.0); the median overall number of rituximab (SC and IV) treatment cycles was 13 (range 2–20) with a median number of 12 rituximab SC administrations (range: 1–19). The median treatment duration was slightly higher in hospitals (18.4 months) compared with practices (16.9 months).

In the DLBCL group, the median duration was 4.6 months (range: 0.3–10.1). The median overall number of treatment cycles was 8 (range 2–8) and patients received a median number of 7 rituximab SC administrations (range: 1–7). No differences between hospitals and practices were observed.

### 3.5 Chemotherapy regimens

The median number of chemotherapy cycles was 6.0 in both groups with a range of 1 to 8 cycles for FL and 2 to 8 cycles for DLBCL patients. Most frequent alterations or dose reductions in the chemotherapy regimen were performed for bendamustine in the FL group (alterations: 27.5%, reductions: 19.2%) and for vincristine in the DLBCL group (alterations: 27.9%, reductions: 25.4%).

Main chemotherapy regimen in the FL group was bendamustine with 166 (67.2%) patients, followed by CHOP with 70 (28.3%) patients. In the DLBCL group, mainly CHOP‐21 or CHOP‐14 protocols were used in 152 (45.2%) and 142 (42.3%) of patients. The predominantly used protocol was CHOP‐14 in hospitals (52% of patients) and CHOP‐21 in practices (55% of patients) (Table 4).

**TABLE 4:** Chemotherapy combination partners at baseline in the FL and DLBCL set, and subgroups by type of site (hospital vs. practice)

FL set Chemotherapy	Total (*N* = 247)	Hospitals (*N* = 104)	Practices (*N* = 143)
Bendamustine	166 (67.2%)	70 (67.3%)	96 (67.1%)
CHOP	70 (28.3%)	34 (32.7%)	36 (25.2%)
CVP	10 (4.0%)	—	10 (7.0%)
R‐CHOP	1 (0.4%)	—	1 (0.7%)

DLBCL set Chemotherapy	Total (*N* = 336)	Hospitals (*N* = 165)	Practices (*N* = 171)
CHOP‐21	152 (45.2%)	58 (35.2%)	94 (55.0%)
CHOP‐14	142 (42.3%)	85 (51.5%)	57 (33.3%)
Other^a^	42 (12.5%)	22 (13.3%)	20 (11.7%)

Abbreviations: CHOEP, cyclophosphamide/doxorubicin/vincristine/prednisone + etoposide; CHOP, cyclophosphamide/doxorubicin/vincristine/prednisone; CHOP‐14, CHOP with a two‐week therapy interval; CHOP‐21, CHOP with a three‐week therapy interval; CVP, cyclophosphamide/vincristine/prednisone; DLBCL, diffuse large B cell lymphoma; FL, follicular lymphoma; R‐CHOP, rituximab + CHOP.

^a^ Mainly mini‐CHOP/CHOEP/bendamustine.

### 3.6 Assessment of patient and nurse satisfaction with rituximab SC

**FIGURE 3:**
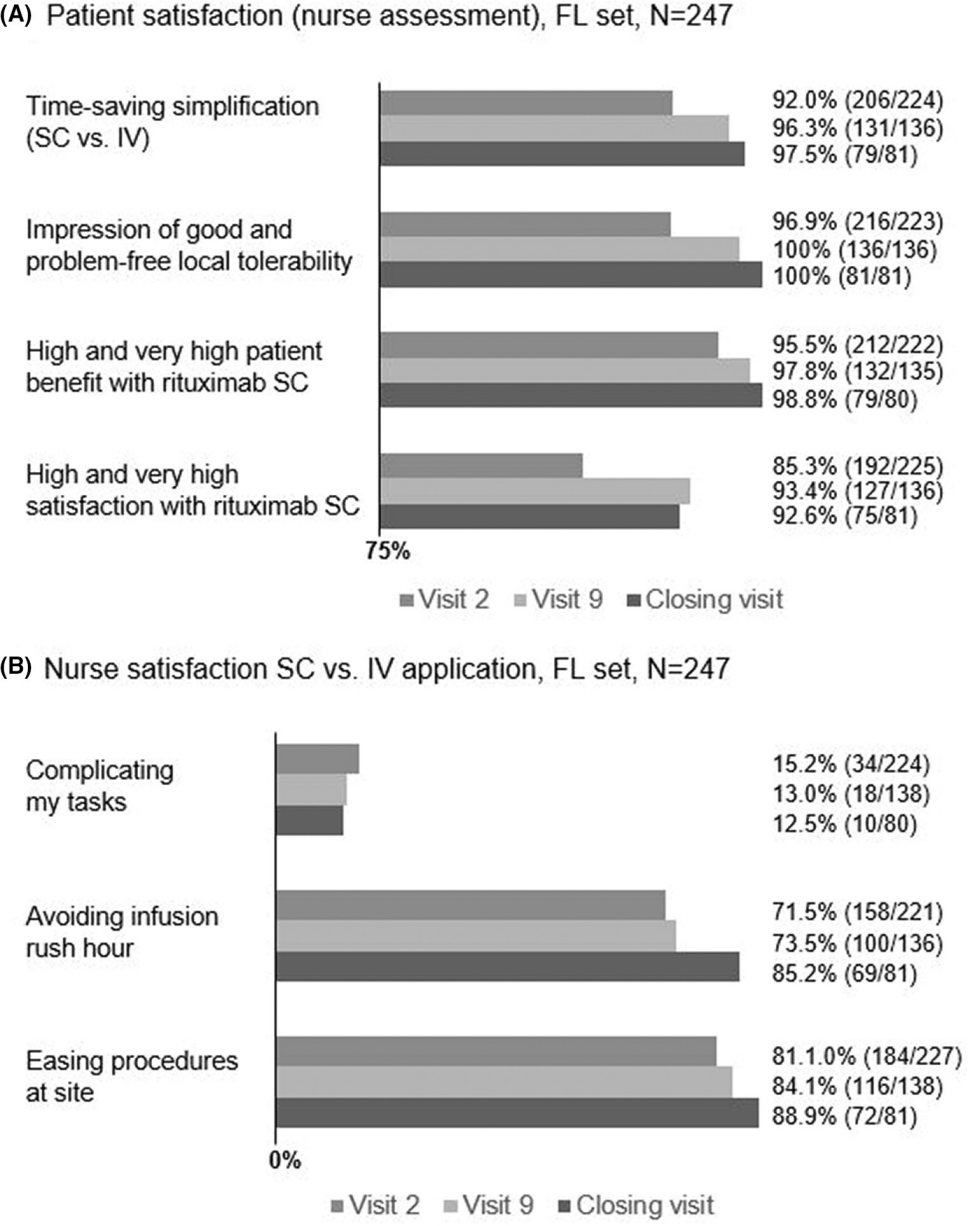
Patient and nurse satisfaction with rituximab SC in the FL set. Patient satisfaction as assessed by the nurse (A) and nurse satisfaction (B) during the study (visit 2, visit 9 and closing visit). FL, follicular lymphoma; IV, intravenous; SC, subcutaneous.

**FIGURE 4:**
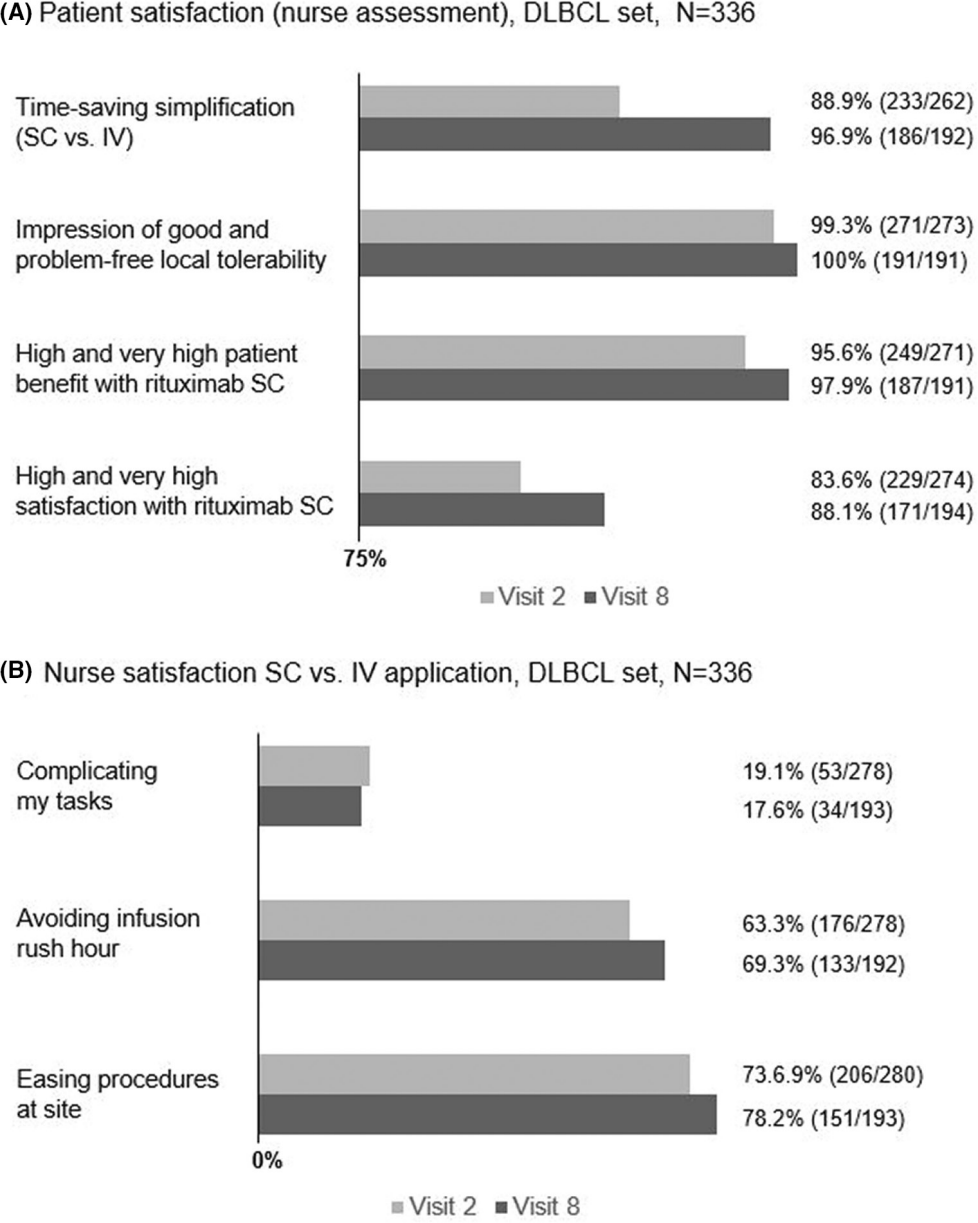
Patient and nurse satisfaction with rituximab SC in the DLBCL set. Patient satisfaction as assessed by the nurse (A) and nurse satisfaction (B) during the study (visit 2 and visit 8). DLBCL, diffuse large B cell lymphoma; IV, intravenous; SC, subcutaneous.

Patient satisfaction as assessed by the study nurse as well as nurse satisfaction during induction (visit 2 to visit 8/9) and maintenance phase for FL patients (visit 9 to closing visit) showed similar results and high acceptance in both the FL (Figure 3) and DLBCL (Figure 4) set. Additionally, nurse satisfaction in applying SC versus IV treatment regarding easing of procedures, avoidance of infusion rush‐hour and less complication of tasks was remarkably higher with rituximab SC in both treatments (Figure 3, Figure 4).

### 3.7 Safety

In the FL group, 228 (92.3%) patients experienced an AE and 83 (33.6%) patients an SAE (Table 5). AEs of CTCAE grade ≥3 were experienced by 112 (45.3%) and SAEs of CTCAE grade ≥3 by 69 (27.9%) patients. Three (1.2%) patients had a fatal AE (grade 5); of these, two patients had grade 5 AEs in the system organ class “cardiac disorders” (i.e., aortic valve stenosis and myocardial infarction) and one patient died from malignant neoplasm progression. None of these events were judged to be related to study treatment.

**TABLE 5:** Summary of adverse events in the FL and DLBCL set including most frequent adverse events, adverse events of CTCAE grade ≥3 and adverse events related to rituximab SC

	FL set (*N* = 247)		DLBCL set (*N* = 336)	
Patients with	Patients *n* (%)	Events *n*	Patients *n* (%)	Events *n*
Any AEs	228 (92.3%)	1511	296 (88.1%)	1787
AEs in ≥10% of patients in any group:				
Leukopenia	63 (25.5%)	89	72 (21.4%)	135
Anemia	29 (11.7%)	38	78 (23.2%)	107
Nausea	53 (21.5%)	69	49 (14.6%)	58
Fatigue	46 (18.6%)	54	57 (17.0%)	66
Neutropenia	22 (8.9%)	33	39 (11.6%)	53
Polyneuropathy	21 (8.5%)	21	36 (10.7%)	38
Cough	25 (10.1%)	32	21 (6.3%)	24
SAEs	83 (33.6%)	144	126 (37.5%)	329
AEs leading to death^a^	3 (1.2%)	3	16 (4.8%)	20
AEs of CTCAE grade ≥3	112 (45.3%)	198	158 (47.0%)	408
AEs of CTCAE grade ≥3 in ≥3% of patients in any group:				
Leukopenia	26 (10.5%)	29	47 (14.0%)	85
Neutropenia	11 (4.5%)	14	30 (8.9%)	39
Anemia	3 (1.2%)	5	16 (4.8%)	20
White blood cell count decreased	3 (1.2%)	3	11 (3.3%)	16
Thrombocytopenia	4 (1.6%)	4	11 (3.3%)	15
Febrile neutropenia	4 (1.6%)	4	13 (3.9%)	14
SAEs of CTCAE grade ≥3	69 (27.9%)	92	109 (32.4%)	233
AEs related to rituximab SC	83 (33.6%)	215	61 (18.2%)	101
AEs related to rituximab SC in ≥3% of patients in any group:				
Injection site erythema	20 (8.1%)	29	3 (0.9%)	3
Skin reaction	10 (4.0%)	15	10 (3.0%)	10
Erythema	13 (5.3%)	19	1 (0.3%)	1
Injection site pain	8 (3.2%)	10	4 (1.2%)	5
SAEs related to rituximab SC	11 (4.5%)	15	19 (5.7%)	21
AEs leading to treatment discontinuation	36 (14.6%)	43	23 (6.8%)	29

Abbreviations: AE, adverse event; CTCAE, Common Terminology Criteria for Adverse Events; DLBCL, diffuse large B cell lymphoma; FL, follicular lymphoma; SAE, serious adverse event; SC, subcutaneous.

^a^ In the entire study, each preferred term for fatal adverse events occurred only once or twice. Two fatal cases, a 72‐year‐old male patient with pulmonary toxicity and an 80‐year‐old male patient with cerebral infarction, were assessed to be causally related to rituximab SC treatment while all other fatal cases were assessed not to be causally related to rituximab SC treatment.

A suspected relationship to rituximab SC was recorded in 83 (33.6%) patients; 11 (4.5%) patients had serious adverse drug reactions (ADRs); 14 (5.7%) patients had grade ≥3 ADRs. AEs resulting in withdrawal/interruption of rituximab SC were observed in 36 (14.6%) patients.

In the DLBCL group, 296 (88.1%) patients experienced an AE and 126 (37.5%) patients an SAE (Table 5). AEs of CTCAE grade ≥3 were experienced by 158 (47.0%) and SAEs of CTCAE grade ≥3 by 109 (32.4%) patients. Sixteen (4.8%) patients had a fatal AE (grade 5), of these two cases, that is, pulmonary toxicity and cerebral infarction, were judged to be causally related to rituximab by the treating physician.

Of the total DLBCL set, 61 (18.2%) patients experienced ADRs related to rituximab. Nineteen (5.7%) patients had serious ADRs or grade ≥3 ADRs, respectively. AEs resulting in withdrawal/interruption of rituximab SC were observed in 23 (6.8%) patients.

In general, fewer AEs were seen as drug related in the DLBCL group than for the FL group (FL 33.6% vs. DLBCL 18.2%), although ADRs with CTCAE grade ≥3 were similar with 5.7% of patients in each group. The proportion of patients with AEs leading to discontinuation was higher in the FL group (FL 14.6% vs. DLBCL 6.8%). The latter may be influenced by the longer treatment duration in the FL set.

Regarding MedDRA system organ classes and preferred terms, the same general body systems—mainly “blood and lymphatic system disorder”—were affected in both groups with slightly different percentages of the single AE terms. Differences were observed in the system organ classes “infections and infestations” (FL 40.1%, DLBCL 29.8%) and “skin and subcutaneous tissue disorders” (FL 32.8%, DLBCL 20.8%).

## 4 DISCUSSION

Extensive clinical experience with intravenously administered rituximab in B cell hematologic malignancies exists, with a global outreach exceeding more than 4 million patients worldwide over 20 years (Roche, data on file not publicly available,^26^). As monotherapy and in combination with chemotherapy, rituximab has prolonged PFS in many patients, and in some indications overall survival, while enjoying a well‐established and manageable safety profile. Originally formulated for IV infusion, the need for a subcutaneous formulation was desirable because IV infusions generally place substantial burden both on patients and healthcare systems, especially in low‐ and middle‐income countries. Additionally, such a formulation would facilitate access to treatment, while freeing up healthcare provider capacity generally.^27^ Accordingly, rituximab SC was developed as a subcutaneous formulation with a systematic, sequential clinical development program. This has demonstrated that rituximab SC achieves non‐inferior serum trough concentrations at fixed doses in patients with NHL and chronic lymphocytic leukemia, with similar efficacy and safety compared with the IV formulation.^27^

The NIS MabSCale sought to confirm the effectiveness and tolerability with patients in both main indications (FL and DLBCL) seen in randomized clinical trials and to supplement the rituximab SC marketing authorization data through data from clinical practice. In this study, the FL set comprised 247 patients and the DLBCL set comprised 336 patients. Regarding baseline, the median age was 65 years (range 26–90 years) in the FL group and 68 years (range 24–90 years) in the DLBCL group, reflecting the disease prevalence under real‐world conditions.

In the following, the MabSCale results are placed in the context of previous clinical trials. It has to be considered, however, that comparability between studies is limited due to the different design (e.g., interventional vs. non‐interventional) and heterogeneous therapeutic regimens. The analysis of the primary variable shows effectiveness of rituximab SC in both indication groups in clinical routine treatment, with CR/CRu rates of 51.4% and 48.5%, respectively. Sensitivity analyses at the end of induction phase and end of study resulted in CR/CRu rates of 34.4% and 37.2% for FL patients. In the DLBCL set, the CR/CRu rate at the end of study was 44.3%. These response rates are comparable with results from the SABRINA trial (FL: CR/CRu rate at EOI 32.2%). In the MabEASE trial^7^ with DLBCL patients, CR/CRu rates at EOI were, however, slightly higher compared with the MabSCale results (50.6% vs. 44.3%).

The overall response (complete and partial) at the end of the study of 85.8% (FL) and 85.4% (DLBCL) agree with previous findings (SABRINA 84.4%).^21^ Different from SABRINA, the main chemotherapy regimen in the FL group of NIS MabSCale was bendamustine with 166/247 (67.2%) patients. The 2‐year PFS rate for the whole group of FL patients was 86.2% comparing favorably with previously published randomized phase III trials.^28^ Therefore, new real‐world evidence was obtained that complemented data from previous randomized controlled trials.

This study confirmed the favorable and well‐known safety profile of rituximab SC. In the FL and DLBCL group, the observed AEs did not vary substantially from results of previous trials. For example, in the FL set of MabSCale (*n* = 247), AEs of any grade were experienced by 92.3% of patients, severe AEs (grade 3–5) by 45.3%, SAEs by 33.6%, and ADRs by 33.6%. By comparison, AEs of any grade were experienced by 96% of patients in the SABRINA trial (*N* = 197 in the SC group), severe AEs (grade 3–5) by 56%, SAEs by 37% and ADRs by 48%.^21^ Thus, the NIS MabSCale, with slightly lower AE incidences did not uncover any significant AEs compared with previous studies, indicating a favorable safety profile of the therapeutic antibody.

As with any NIS, the study design had some limitations. Clinical assessments were not mandatory, and as such, the type, frequency, method, and potential confirmation of a finding were solely based on routine medical practice. Nevertheless, care was taken to collect and report data in a consistent way, avoiding possible bias. Remarkably, a considerable number of patients did not complete the two‐year maintenance phase in the FL set, had no final staging or were lost to follow‐up, thus affecting the assessment of the primary and secondary endpoints. Moreover, data quality was affected by partly incomplete data documentation, particularly at the sites that were closed early.

In order to investigate treatment modalities in general and shed light on potential differences between hospitals and private practices, a post hoc analysis was carried out. A noteworthy difference was observed for the predominantly used chemotherapy regimen (hospitals: 52% of patients treated with CHOP‐14; practices: 55% of patients treated with CHOP‐21). Furthermore, our data show an unexpected treatment pattern in FL patients with limited stage I/II disease accounting for about one quarter of the study cohort (Table 2). These patients may be cured with radio(immuno)therapy^29^, ^30^ but received chemoimmunotherapy with palliative intent, which is in line with findings from the NCLS study reported by Nastoupil et al.^31^ and the recently published RIM trial.^32^ Interestingly, in our study, FL patients with limited disease were more prevalent in the private practice (30.8%) as compared to the hospital treatment setting (20.2%). Our real‐world results together with findings from the RIM trial may suggest a shift from radiotherapy as recommended in current guidelines to early systemic treatment in this patient subgroup.

Previous studies have highlighted advantages in a SC application of rituximab over IV administration. Accordingly, assessment of patient and nurse satisfaction was a secondary objective of this study. The results clearly indicated that the vast majority of patients (>90% in both indication sets) regarded the SC application as highly beneficial. For example, patient satisfaction with the rituximab SC route vs. IV was 96.3% in the FL group (visit 9) and 96.9% for DLBCL (visit 8) regarding time‐saving simplification. These results were superior to patient satisfaction reported in the previous PrefMab study,^22^ where 81% of patients preferred the rituximab SC route compared with IV administration at cycle 8.

For patients with poor venous access, rituximab SC provides a convenient alternative route of administration, while reducing the number of uncomfortable catheter implantations or the risk of catheter‐related sepsis. Subcutaneous application might be accompanied by an increased rate of local reactions at the injection site, as reported in some clinical studies. These events were largely manageable and corresponded to the expected changes when switching from IV to SC administration. As treatment progresses, such reactions usually decrease in intensity and frequency.^27^ In the NIS MabSCale, the incidence of injection site pain or erythema was accordingly low.

## AUTHOR CONTRIBUTIONS

Jan Dürig: Conceptualization, investigation, formal analysis, writing and review of the manuscript. Jens Uhlig: Investigation, writing and review of the manuscript. Anke Gerhardt: Investigation, writing and review of the manuscript. Markus Ritter: Investigation, writing and review of the manuscript. Gunnar Hapke: Investigation, writing and review of the manuscript. Jörg Heßling: Investigation, writing and review of the manuscript. Peter Staib: Investigation, writing and review of the manuscript. Frieder Wolff: Conceptualization, investigation, formal analysis, writing and review of the manuscript. Katja Krumm: Conceptualization, investigation, formal analysis, writing and review of the manuscript. Ludwig Fischer von Weikersthal: Investigation, writing and review of the manuscript.

## FUNDING INFORMATION

Roche Pharma AG, Grenzach‐Wyhlen, Germany provided financial support for this research. Roche was involved in design and conduct of the study; collection, management, analysis, and interpretation of data; and preparation, review, and approval of the manuscript.

## CONFLICT OF INTEREST

J. Dürig reports membership of advisory boards of Abbvie, Amgen, BMS, Celgene, Janssen, Roche and Takeda; speaker's fees from Abbvie, Amgen, Astra Zeneca, BMS, Celgene, Janssen, Roche and Takeda; and travel support from Celgene and Janssen. J. Uhlig reports membership of advisory boards or consulting and participation in workshops of Amgen, Bayer, Beigene, Boehringer‐Ingelheim, Bristol‐Myers Squibb, Celgene, Janssen‐Cilag, Merck, MSD, Novartis, Roche, Sanofi and Servier. A. Gerhardt reports membership of advisory boards of Amgen, Janssen and Pfizer; congress support from Behring, Celgene, Janssen and Novartis; and honoraria from Janssen. M. Ritter reports membership of an advisory board of Boehringer Ingelheim and congress support from Novartis. G. Happke reports consulting of Abbvie, Janssen and Roche; and grants from Biontech, BMS, Gilead, Ipsen, Merck, MSD and Roche. P. Staib reports grants, personal fees, non‐financial support, and research support from Abbvie, Amgen, Celgene, Gilead, Janssen‐Cilag, Novartis, Pfizer and Roche. F. Wolff is an external business partner of Roche. K. Krumm is an employee of Roche. L. Fischer von Weikersthal reports speaker's fees from Lily, Novartis and Pierre Fabre. J. Heßling reports no conflict of interest.

## ETHICS APPROVAL STATEMENT

An independent ethics committee (Ethics Committee of the Landesärztekammer Baden‐Württemberg, Germany) approved the study on 03 December 2013. Every participating patient had provided signed informed consent according to the regulatory and legal requirements. The study was registered under clinicaltrials.gov identifier NCT02240316.

## Supporting information


Figure S1

Figure S2

Figure S3

Table S1

## Data Availability

Qualified researchers may request access to individual patient level data through the clinical study data request platform (https://vivli.org/). Further details on Roche's criteria for eligible studies are available here (https://vivli.org/members/ourmembers/). For further details on Roche's Global Policy on the Sharing of Clinical Information and how to request access to related clinical study documents, see here (https://www.roche.com/research_and_development/who_we_are_how_we_work/clinical_trials/our_commitment_to_data_sharing.htm).
