# Supplementary figures and images for: Subcutaneous rituximab in patients with diffuse large B cell lymphoma and follicular lymphoma: Final results of the non‐interventional study MabSCale
Source: Cancer Med. 2022 Aug 26;12(3):2739–51. doi: 10.1002/cam4.5160 (PMC9939131; doi:10.1002/cam4.5160)

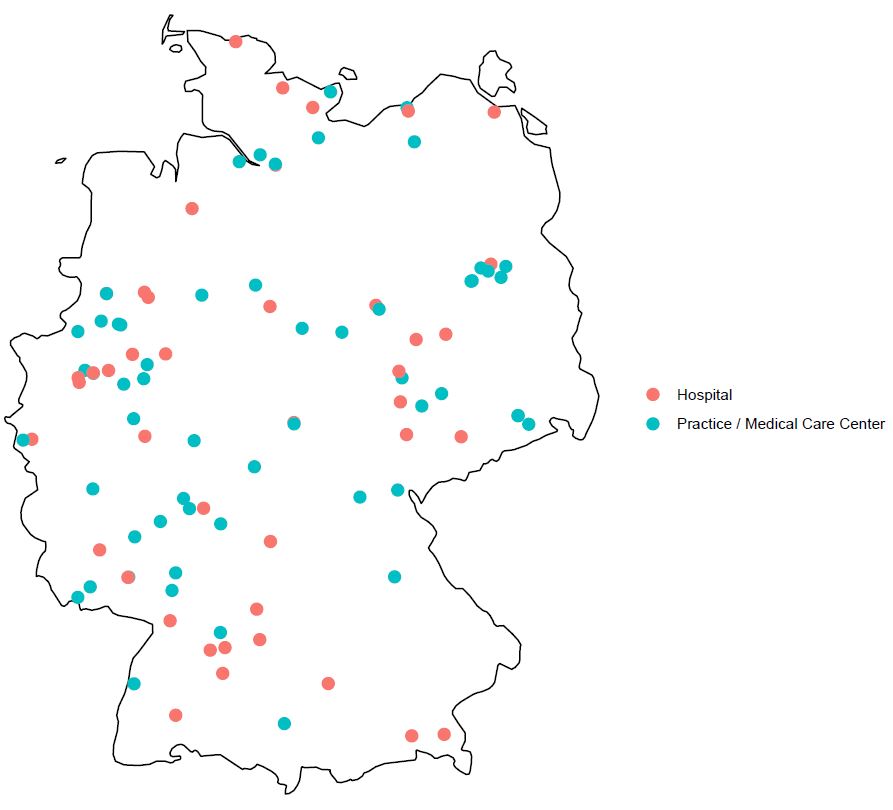


**Figure S1: Distribution of study sites in Germany**

Supplement: Supplementary file 1 — Figure S1 [file CAM4-12-2739-s003.docx]
