# Supplementary material for: Subcutaneous rituximab in patients with diffuse large B cell lymphoma and follicular lymphoma: Final results of the non‐interventional study MabSCale
Source: Cancer Med. 2022 Aug 26;12(3):2739–51. doi: 10.1002/cam4.5160 (PMC9939131; doi:10.1002/cam4.5160)
Supplement: Supplementary file 2 — Figure S2 [file CAM4-12-2739-s004.docx]

Excluded: N=13

- Violation of inclusion criterion 2 (untreated FL or DLBCL), N=12
- Violation of inclusion criterion 5 (first-line treatment with rituximab), N=1

Excluded during Data Review Meeting: N=8

- Treatment without informed consent, N=4
- Not permitted previous treatment, N=4

**Screened patients: N=689**

(Informed consent signed)

**Treated patients: N=583**

(At least one dose of rituximab SC)

**Enrolled patients: N=668**

(Inclusion and exclusion criteria met)

Excluded: N=85

- Patients without rituximab SC treatment, N=85

**FL set:**

**N=247**

Main reasons for discontinuation:

- Discontinuation of rituximab by physician: FL N=37, DLBCL N=15
- Discontinuation by patient: FL N=14, DLBCL N=6
- Disease progression: FL N=19, DLBCL N=16
- Other reasons: FL N=28, DLBCL N=25
- Death^†^: FL N=3, DLBCL N=12

^†^Data processed as per end of observation visit, different from AE grade 5 assessment

**DLBCL set:**

**N=336**

**242 completed the study**

**126 completed the study**

**Figure S2: Patient disposition.** Abbreviations: AE, adverse event; DLBCL, diffuse large B cell lymphoma; FL, follicular lymphoma; SC, subcutaneous.
