# Supplementary material for: Subcutaneous rituximab in patients with diffuse large B cell lymphoma and follicular lymphoma: Final results of the non‐interventional study MabSCale
Source: Cancer Med. 2022 Aug 26;12(3):2739–51. doi: 10.1002/cam4.5160 (PMC9939131; doi:10.1002/cam4.5160)
Supplement: Supplementary file 3 — Figure S3 [file CAM4-12-2739-s001.docx]

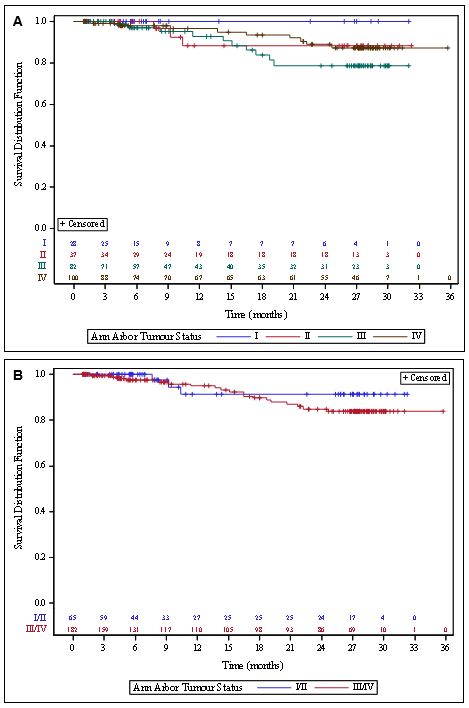


**Figure S3: Kaplan-Meier plot of PFS in the FL set by Ann Arbor tumor status I, II, III and IV**

(A) Ann Arbor tumor status I vs. II vs. III vs. IV. (B) Ann Arbor tumor status I/II vs. III/IV. PFS was defined as time from first visit until progression or death. Patients without event were censored at their last observation (last visit or discontinuation). Abbreviations: FL, follicular lymphoma; PFS, progression-free survival
