# Supplementary material for: Subcutaneous rituximab in patients with diffuse large B cell lymphoma and follicular lymphoma: Final results of the non‐interventional study MabSCale
Source: Cancer Med. 2022 Aug 26;12(3):2739–51. doi: 10.1002/cam4.5160 (PMC9939131; doi:10.1002/cam4.5160)
Supplement: Supplementary file 4 — Table S1 [file CAM4-12-2739-s002.docx]

**Table S1: CR/CRu rate by subgroups in the FL and DLBCL set**

|  |  | **CR/CRu achieved** | |
| --- | --- | --- | --- |
| **Subgroup** |  | **FL set** | **DLBCL set** |
| Age category [years] | | | |
| <65 (FL), <60 (DLBCL) | n | 113 | 90 |
|  | Rate [95% CI] | 62.8 [53.6; 71.2] | 56.7 [46.4; 66.4] |
| ≥65 - <75 (FL), ≥60 - <80 (DLBCL) | n | 80 | 197 |
|  | Rate [95% CI] | 45.0 [34.6; 55.9] | 46.7 [39.9; 53.7] |
| ≥75 (FL), ≥80 (DLBCL) | n | 54 | 49 |
|  | Rate [95% CI] | 37.0 [25.4; 50.4] | 40.8 [28.2; 54.8] |
| Gender | | | |
| Men | n | 121 | 194 |
|  | Rate [95% CI] | 51.2 [42.4; 60.0] | 47.4 [40.5; 54.4] |
| Women | n | 126 | 142 |
|  | Rate [95% CI] | 51.6 [42.9; 60.1] | 50.0 [41.9; 58.1] |
| ECOG performance status | | | |
| 0 | n | 155 | 163 |
|  | Rate [95% CI] | 56.1 [48.3; 63.7] | 54.0 [46.3; 61.5] |
| 1 | n | 76 | 139 |
|  | Rate [95% CI] | 50.0 [39.0; 61.0] | 46.0 [38.0; 54.3] |
| 2 | n | 8 | 21 |
|  | Rate [95% CI] | NA | 9.5 [2.7; 28.9] |
| 3 | n | 3 | 1 |
|  | Rate [95% CI] | NA | 100.0 [20.7; 100.0] |
| Ann Arbor tumor status | | | |
| I | n | 28 | 96 |
|  | Rate [95% CI] | 57.1 [39.1; 73.5] | 54.2 [44.2; 63.8] |
| II | n | 37 | 83 |
|  | Rate [95% CI] | 51.4 [35.9; 66.6] | 53.0 [42.4; 63.4] |
| III | n | 82 | 62 |
|  | Rate [95% CI] | 51.2 [40.6; 61.7] | 54.8 [42.5; 66.6] |
| IV | n | 100 | 94 |
|  | Rate [95% CI] | 50.0 [40.4; 59.6] | 34.0 [25.3; 44.1] |
| Abbreviations: CR, complete remission; CRu, complete remission unconfirmed; DLBCL, diffuse large B cell lymphoma; ECOG, Eastern Cooperative Oncology Group; FL, follicular lymphoma; NA, not applicable | | | |
